# Staphylococcal protein A modulates inflammation by inducing interferon signaling in human nasal epithelial cells

**DOI:** 10.1007/s00011-022-01656-1

**Published:** 2022-12-17

**Authors:** Hua Hu, Sha Liu, Karen Hon, Alkis J. Psaltis, Peter John Wormald, Sarah Vreugde

**Affiliations:** 1 Department of Surgery-Otolaryngology Head and Neck Surgery, Basil Hetzel Institute for Translational Health Research, Central Adelaide Local Health Network, Woodville, SA Australia; 2 Department of Otolaryngology Head & Neck Surgery, Adelaide Medical School, The University of Adelaide, Adelaide, SA Australia; 3 Department of Otolaryngology, Head and Neck Surgery, Shanghai General Hospital, Shanghai Jiaotong University, Shanghai, China

**Keywords:** IFNGR1, Jak2, *Staphylococcus aureus*, Staphylococcal protein A, Type II IFN-Jak-STAT pathway

## Abstract

**Objective and design:**

*Staphylococcus aureus* (*S. aureus*) is one of the leading causes of human respiratory tract infections. The function of Staphylococcal protein A (SpA), expressed on the *S. aureus* bacterial membrane and released in the environment, on human nasal epithelial cells (HNECs) have not been fully elucidated. In this study, we tested the SpA expression in *S. aureus* from chronic rhinosinusitis patients and investigated the effects of SpA on HNECs inflammation through Interferon Gamma Receptor 1(IFNGR1)/phosphorylated Janus Kinase 2 (p-JAK2) pathway.

**Methods:**

RNA profiling was performed to investigate inflammatory activation in a *S. aureus* chronic rhinosinusitis (CRS) mouse model. SpA release by *S. aureus* clinical isolates was determined using ELISA. The effect of purified SpA and SpA enriched conditioned media from *S. aureus* clinical isolates on HNECs cytotoxicity, apoptosis and release of inflammatory cytokines was evaluated using lactate dehydrogenase assays, and flow cytometry. SpA dependent IFNGR1 and p-JAK2 expression were assessed by qPCR, immunofluorescence and western blot in HNECs.

**Results:**

49 genes were significantly induced in *S. aureus* CRS mice indicative of activation of interferon signaling. SpA release was significantly higher in *S. aureus* clinical isolates from chronic rhinosinusitis with nasal polyps (CRSwNP) patients. Purified SpA significantly increased IFNGR1 mRNA and protein expression in HNECs. SpA induced cytotoxic effects and induced the release of Interleukin-6 (IL-6) and IL-8 in an IFNGR1 dependent way.

**Conclusion:**

SpA induces interferon signaling through activation of the IFNGR1-JAK-2 pathway, which provides an understanding of how *S. aureus* SpA affects the inflammatory process in the upper airways.

**Supplementary Information:**

The online version contains supplementary material available at 10.1007/s00011-022-01656-1.

## Introduction

*S. aureus* is a common pathogenic bacterium that causes nosocomial and community-related infection, as well as various invasive infections including bacteremia, osteomyelitis and septic arthritis [1–3]. Currently, antibiotic therapy is the primary method of treating *S. aureus* infections. The bacteriostatic or bactericidal action of antibiotics has been shown to result in selective pressure for bacteria, promoting the emergence of drug-resistant *S. aureus* strains [4]. Amongst various bacterial and fungal pathogens, the prevalence of methicillin-resistant *Staphylococcus aureus* (MRSA) strains has risen rapidly in the last few decades [5, 6]. Infections with MRSA have a high mortality rate compared to those caused by Methicillin Sensitive *S. aureus* (MSSA) strains. Consequently, there is an urgent need for the development of new strategies to combat infections with antibiotic resistant pathogens including MRSA [7, 8].

The main pathogenic mechanisms of *S. aureus* are linked to the secretion of various virulence factors including immune evasion factors that allow *S. aureus* to escape the host immune system surveillance and response [9–12]. Hence, more recent therapeutic strategies are aimed at reducing the virulence of bacteria by selectively suppressing the expression and transmission of toxins and bacterial adhesion and immune evasion factors. Rather than directly killing or suppressing the viability of the bacteria using antibiotics, such strategies have recently gained attention for their relative lack of selective pressure on the targeted bacteria resulting in a reduction in the formation of antibiotic resistant strains [13, 14]. However, for such strategies to be effective, it is critical to identify the key virulence factors and their signaling pathways.

Amongst the many virulence factors produced by *S. aureus*, Staphylococcal protein A (SpA) is expressed by most clinical isolates and enables *S. aureus* to evade host immune responses. SpA is present as a *S. aureus* membrane protein, consisting of 5 homologous domains (E, D, A, B, C) that are linked by several polymorphic Xr-regions [15]. Each homologous domain of SpA can bind to the Fc portion of IgG and Antibody-binding fragments (Fab) domain of V_H_3 with high affinity [16]. Indeed, SpA’s binding with IgG Fc segments inhibits phagocytic processes via the consumption of immunoglobulins reducing their binding with phagocytic Fcγ receptors [17]. Furthermore, in a *S. aureus* sepsis mouse model, an SpA mutant *S. aureus* showed lower virulence linked to a reduced binding of the lgG Fc segment to mutant SpA thereby reducing *S. aureus* phagocytosis [17–19].

Additionally, SpA can initiate the apoptosis of cells expressing the H chain variable region (VH) of human Abs, specifically V_H_3 family expressing B-cells through a T-cell-independent pathway [20]. This impacts 2 B cell subpopulations, B-1a and Marginal Zone (MZ) B-cells [21]. B-1a cells are known for secreting a large number of antibodies, which have significant protective effects against different pathogenic bacteria [22], whilst MZ B cells express different antigen-binding profiles—which mainly contribute to the T-cell-independent immune response against the non-protein antigens of infectious bacteria [21]. SpA is thus regarded as a superantigen that destroys the host innate immune response by clearing B-1a cells and MZ B cells, that are known to exert innate immune like functions and have important roles in the first-line defense against pathogens [23, 24].

The role of the type I and type II interferon (IFN) pathway in the protection against viral infections is well established. Type I IFNs consist of structurally similar cytokines which include the subtypes of IFN-α and IFN-β. These cytokines signal through the same receptor, type I IFN receptor (IFNAR), and are produced early on during a viral infection. They are essential for activating the antiviral innate immune response and act in concert with type II IFN. Type II IFN, known as IFN-γ, signals through a different receptor, the IFN-γ receptor (IFNGR), composed of IFNGR1 and IFNGR2 subunits, and has effects that are independent from type I IFN. IFN-γ promotes antiviral immunity through its regulatory effects on the innate immune response and links the innate and adaptive immune response [25]. Recent evidence has shown that interferon signaling is not isolated to viral stimuli but can be produced during various inflammatory insults. However, its role in bacterial infections is less well defined. *S. aureus* SpA has been shown to activate type I IFN signaling [26] and type I interferon responses have recently been shown to contribute to the pathophysiology of eosinophilic chronic rhinosinusitis (CRS) [27]. In a mouse lung infection model, *S. aureus-*dependent induction of the type I IFN cascade differed between clinical isolates and correlated with the intensity of the inflammatory response and tissue damage [28]. However, the potential involvement of type II IFN signaling contributing to such responses in CRS patients remains unknown.

In this study, we have used *S. aureus* clinical isolates from CRS patients to determine their SpA secretion and their differential potential for SpA induced type II IFN signaling in primary human nasal epithelial cell cultures. We have also investigated the potential for *S. aureus* dependent activation of the type II interferon signaling pathway in the context of chronic rhinosinusitis *in vivo*.

## Materials and methods

### Cells, bacteria and reagents

The study was approved by the Central Adelaide Local Health Network Human Research Ethics Committee (HREC/15/TQEH/132). All patients gave written informed consent before tissue collection. Primary Human Nasal Epithelial cells (HNECs) and *S. aureus* clinical isolates were obtained from patients undergoing endoscopic sinus surgery (ESS) for chronic rhinosinusitis (CRS) or septoplasty (controls). Controls had no clinical or endoscopic features of CRS. CRS patients met the diagnostic criteria for this condition as set out in the EPOS 2012 document and were classified as CRS with polyps (CRSwNP) and without polyps (CRSsNP) based on their endoscopic evaluation [29]. *S. aureus* clinical isolates were isolated by an independent pathology laboratory (Adelaide Pathology Partners, Adelaide, Australia) and stored at – 80°C in glycerol stocks until use. *S. aureus* ATCC51650 was obtained from the American Type Culture Collection (ATCC, Manassas, USA). Purified SpA (≥95% purity) was purchased from Sigma-Aldrich (Sigma-Aldrich, St. Louis, USA).

### SpA enzyme-linked immunosorbent assays (ELISA) assay

*S. aureus* clinical isolates were cultured in Tryptic Soy Broth (TSB) (Thermo Fisher Scientific; Waltham, USA) for 24 h at 37 ℃. The planktonic samples were centrifuged at 3000 rpm for 10 min. The supernatant was then collected and filtered using a 0.22 micrometer filter (Pall Corporation, New York, USA). SpA protein content in *S. aureus* planktonic bacteria was determined using a SpA ELISA kit (Abcam, Cambridge, UK) according to the manufacturer’s instructions. The optical density was tested at 450 nm and SpA content was determined based on the standard curve prepared alongside. Each experiment was performed in duplicate.

### CRS mouse model

All experimental procedures using the CRS mouse model were approved by the Institutional animal care and use committee of Shanghai Jiaotong University (2019SQ046). 10–12 weeks Specific Pathogen Free (SPF) C57BL/6J mice (*n*=12) were obtained from Shanghai Laboratory animal center (Shanghai, China) and housed in a 12 h light/dark cycle with soft food and tap water provided continuously ad libitum. The CRS mouse model was modified from the method described by Jacob et al [30]. Briefly, for the CRS group (*n* = 6), the right bony anterior naris was exposed by making an incision from the right snout to the nasal dorsum. A 5-mm length Merocel nasal pack (Merocel, USA), soaked with 20 μl *S. aureus* ATCC 51650 (10^8^ CFU in 0.9% saline) overnight culture was inserted into the mouse’s right nasal cavity and the incision was closed. Mice were then kept for 3 months to allow the induction of CRS. The control group (*n* = 6) mice underwent the same surgical procedure without Merocel nasal packing and *S. aureus* inoculation.

### Histological analysis

The mouse nasal tissues were harvested. Nasal tissue was randomly selected to be stored in RNAlater (Sigma-Aldrich, St. Louis, USA) first and then liquid nitrogen until processed for RNA extraction (*n*=3 for each of CRS and control mice) or they were fixed in 10% neutral buffered formalin (*n* = 3 for each of CRS and control mice), decalcified in 10% EDTA solution for 2 weeks and embedded in paraffin blocks. Paraffin blocks were then cut at 6 μm thickness, put onto glass slides and stained with hematoxylin and eosin. Then, sections were imaged with a Leica light microscope system (Leica Microsystems GmbH, Wetzlar, Germany).

### RNA profiling of mouse nasal tissue

In brief, total RNA from mice nasal tissue was extracted using TAKARA RNAiso plus kit (Takara Bio, Shiga, JAPAN) following the manufacturer’s instructions and RNA integrity was checked by an agilent bioanalyzer 2001 (Agilent technologies, Santa Clara, CA, USA). Following this, the total RNA was treated with RNase-Free DNase (Qiagen, GmBH, Germany). For RNA profiling, the Agilent Whole Mouse Genome Microarray 4×44K was used according to the manufacturer’s instruction with 100 ng RNA (RNA integrity number (RIN)> = 7). The samples were hybridized for 20 h at 55 °C and then were scanned using Agilent DNA Microarray Scanner BA. David v6.8 online software (david.abcc.ncifcrf.gov/) was used to perform Kyoto Encyclopedia of Genes and Genomes (KEGG) Pathway analysis. Mice nasal tissue harvested from the control group underwent identical procedures and was used as control.

### Bacterial cell culture and planktonic supernatant collection.

*S. aureus* clinical isolates were plated from frozen glycerol stocks onto 1.5% Tryptone Soya agar (TSA) plates at 37 ℃ overnight. Colonies were picked and 0.5 McFarland unit suspension was adjusted with 0.9% NaCl. The suspension was diluted 1:100 in TSB and incubated at 37 ℃ for 24 h. OD 600 was measured to monitor bacterial growth. The bacterial cultures were then centrifuged at 3000 rpm for 10 min. The planktonic supernatant was collected and filtered using 0.22 micrometer syringe filter (Pall Corporation, San Diego, USA) to remove bacterial cells.

### HNECs culture experiments

Primary HNECs were harvested from nasal mucosa by gentle brushing. Extracted cells were suspended in PneumaCultTM- Ex Plus medium (StemCell Technologies, Vancouver, Canada).

Macrophages were removed by treating the cells with anti-CD68 (Dako, Glostrup, Denmark) coated petri dishes for 20 min at 37 ℃. HNECs were maintained with PneumaCultTM- Ex Plus medium in collagen coated flasks (Thermo Scientific, Walthman, MA, USA) at 37 °C with 5% CO_2_ until confluence. 5*10^5^ cells were seeded in collagen coated 24 well plates (Corning, NY, USA) and 8 well chamber slides (Corning, NY, USA) respectively. Cells were treated with 5% bacterial planktonic supernatants for different times followed by RNA extraction, immunofluorescence staining or protein extraction as described below. Cells treated with purified SpA (50 μg /ml) and 5% TSB were used as positive and negative control, respectively.

### RNA extraction

Primary HNECs in 24 well plates were treated with 5% bacterial planktonic supernatants for 4 hours. HNECs media was then removed and cells washed 3 times with PBS gently. HNECs were harvested using cell scrapers (Sigma-Aldrich, St. Louis, USA) and transferred to an Eppendorf tube. Total RNA from HNECs was extracted using a PureLink® RNA Mini Kit (Life technologies; Mulgrave, VIC, Australia), and DNase digestion was performed to remove genomic DNA using the Purelink DNase set (Life Technologies; Mulgrave, VIC, Australia). The extraction process was done in accordance with the manufacturer’s instructions. Total RNA was quantified by measuring the absorbance at 260nm (A260) using a NanoDropTM 2000 spectrophotometer (Thermo Scientific, Scoresby, VIC, Australia). The purity was estimated via the 260/280 absorbance ratio (A260/A280>2.0 for pure RNA) and a denaturing agarose gel electrophoresis of RNA was performed to confirm the integrity of RNA for further qPCR.

### qPCR

Relative expression of the selected genes was tested using reverse Transcription System (Qiagen) and Taqman qPCR Mixes (Thermo Fisher Scientific, Waltham, MA, USA) and qPCR was carried out according to manufacturer’s instructions. The Biorad CFX96 PCR System (Biorad) was used to detect the reactions. The procedure was: 50 °C for 2 min incubation; 95 °C for 10 min; 95 °C for 15 s, and 60°C for 1 min with 45 cycles. Relative quantification of the gene expression level was presented using the comparative Ct method (2-ΔCt). The primers used were obtained from Thermo Fisher Scientific (Waltham, MA, USA):

GAPDH: (Assay ID: Hs02758991_g1, Thermo Fisher Scientific, Waltham, MA, USA)

INFGR1: (Assay ID: Hs00988304_m1, Thermo Fisher Scientific, Waltham, MA, USA)

### Immunofluorescence staining

Primary HNECs (8 well chamber slides) were treated for 24 h and washed 3 times with PBS gently. Primary HNECs were then fixed with 2.5% formaldehyde, permeabilized with 0.1% Triton X-100 in PBS for 10 min and blocked with serum-free blocker for 1 h (Dako, Glostrup, Denmark). HNECs were incubated with 10ug/ml of Goat monoclonal anti-human IFNGR1 antibody (Invitrogen, Carlsbad, USA) at 4 °C overnight. After washing with TBST 3 times, 1:200 diluted anti-goat Alexa Fluor-488 conjugated secondary antibody (Jackson Immunoresearch Laboratories, West Grove, PA, USA) was added and incubated 1 h at RT followed by DAPI (Sigma-Aldrich, St. Louis, USA) staining for 15 min. After washing with PBS 3 times, a drop of anti-fade mounting medium (Dako, Glostrup, Denmark) was added before cover slipping. Samples were visualized by using an LSM700 confocal laser scanning microscope (Carl-Zeiss, Oberkochen, Germany).

### Western blot

Primary HNECs in 24 well plates were treated for 6 h. HNECs media was then removed and cells were washed 3 times with cold PBS gently. HNECs protein was extracted using Radioimmunoprecipitation assay (RIPA) buffer (Sigma). 100ul cold RIPA buffer was added to the cells and was kept on ice for 5 min. Cell lysates were collected and transferred to a microcentrifuge tube and centrifuged at 14,000 × g for 15 min. The supernatant was transferred to a new tube for further analysis. Protein concentration was determined by Bradford assay kit (Bio-Rad, Hercules, CA, USA). Samples were denatured with 2x Laemmli Sample Buffer (Bio-Rad) at 95 ℃ for 15 min. Aliquots of 15μg total protein were added to Criterion TGX Gel (Bio-Rad) followed by electrophoresis at 300 voltage for 20 min. The samples were transferred to polyvinylidene difluoride (PVDF) membranes (Invitrogen, Carlsbad, USA) by application of 10V for 2 hours. Membranes were washed twice with TBST and blocked with 5% non-fat milk blocking buffer for 1 h at RT. The membrane was incubated with primary antibodies at 4 ℃ overnight. Membranes were washed and incubated with corresponding secondary antibodies for 1 h at room temperature. Images were visualized using Pierce^TM^ ECL solution (Thermo Fisher Scientific, Waltham, MA, USA) and ChemiDoc™ MP (Bio-Rad). Primary antibodies used were Goat anti-human IFNGR1 (1:2000, Invitrogen, USA), Rabbit anti-human p-JAK2 (1:2000, Abcam, Cambridge, UK), Rabbit anti-human profilin 1 (0.5μg/ml, Thermo Fisher Scientific, Waltham, MA, USA); Secondary antibodies were Horseradish peroxidase (HRP)-conjugated anti-goat antibody (1:2000, Santa Cruz Biotechnology Inc, CA, USA), Anti-rabbit antibody (1:10000, Abcam, Cambridge, UK).

### Primary HNECs IFNGR1 gene knockout

Lipofectamine™ 3000 Transfection Reagent (Thermo Fisher Scientific, Waltham, MA, USA) and IFNGR1 siRNAs (siRNA ID106090; Thermo Fisher Scientific, Waltham, MA, USA) were used to knockout IFNGR1 according to the manufacturer’s instructions. Briefly, HNECs were seeded in collagen coated 24-wells plates. Lipofectamine™ 3000 Reagent and siRNA were diluted in Opti-MEM™ (0.75 μl in 25 μl and 1 μg in 50 μl), and then mixed in a 1:1 ratio and incubated for 15 min at RT. The mixture was added onto cells and incubated for 4 days. HNEC proteins were harvested followed by Western blot as above. Lipofectamine™ 3000 Reagent without IFNGR1 siRNAs was used as control.

### Cytotoxicity assay

Cytotoxicity of HNECs was measured using an LDH release kit (Promega, Madison, WI, USA) following the manufacturer’s instructions. Briefly, HNECs in 24-well plates were treated with 5% bacterial planktonic supernatants and purified SpA for 24 hours. The supernatant medium was collected and centrifuged. 50 μL of the medium from each well was transferred to a new plate, and 50 μL of LDH reagent was added to the supernatant and incubated for 30 min in the dark at room temperature (RT). 10% Triton X-100 in medium was used as positive control and 5% tryptic soy broth (TSB) in medium was used as negative control. Absorbance from each well was read using a microplate reader at 490 nm (BMG Labtech, Ortenberg, Germany), and relative viability was calculated relative to the LDH levels of negative controls (untreated cells) and positive controls.

### IL-6 and IL-8 ELISA assay

Interleukin-6 (IL-6) and Interleukin-8 (IL-8) protein levels were determined with IL-6 and IL-8 enzyme-linked immunosorbent assay (ELISA) kits (BD Biosciences, Franklin Lakes, NJ, USA), according to the manufacturer’s instructions after treatment of HNECs with 5% bacterial planktonic supernatants and purified SpA or control for 24 hours. All measurements were performed in duplicate. The optical density (OD) was measured at 450 nm and protein content determined using the standard curve prepared for each assay.

### Apoptosis assay

APC Annexin V Apoptosis Detection Kit with PI (Biolegend, San Diego, CA, USA) was applied to quantify apoptosis according to the manual. In brief, HNECs were seeded in collagen coated 24-well plates. Cells were then harvested after 24 h treatment with 5% bacterial planktonic supernatant and purified SpA and counted. HNECs were stained with Annexin V-APC and PI in the dark for 15 min at RT, followed by flow cytometry analysis using a FACS canto II (BD Biosciences, San Jose, CA, USA) to quantify the apoptosis frequency of cells. Cell stained with both Annexin V-APC and PI were counted as apoptotic cells. Cells without staining and cells stained with Annexin V-APC or PI only were used as control.

### Statistical analysis

Data were expressed as mean ± SE. The GraphPad Prism (GraphPad Prism version 9.00, GraphPad Software, La Jolla, CA, USA) was used for statistical analysis. Comparisons between different groups were made by one way ANOVA with post-hoc Tukey’s multiple comparison test. *P* < 0.05 was considered to indicate a statistically significant difference. David v6.8 online software (david.abcc.ncifcrf.gov/) was used to perform Kyoto Encyclopedia of Genes and Genomes (KEGG) Pathway analysis for the mouse experiments, and Student's t-test was applied to determine the gene expression fold change.

## Results

### *S. aureus* induced IFN signaling in a CRS mouse model

To understand the potential of *S. aureus* chronic sinonasal infection to induce inflammation, a *S. aureus* CRS mouse model was used. Histopathology of mouse sinonasal mucosa showed evidence of severe inflammation with a narrowing of the nasal airway and structural changes in the mucosal cytoarchitecture with ciliary denudation, epithelial dysplasia and inflammatory cell infiltration (Supplementary Figure 1). Gene expression profiling showed differential expression of various genes involved in inflammation including an induction of Interferon gamma (*Ifng*, 3.9 fold, *p* = 0.04), Interferon gamma receptor 1 (*Ifngr1*, 2.8 fold, *p* < 0.0001), Interferon gamma receptor 2 (*Ifgr2*, 3 fold, *p* = 0.0007), Janus kinase 1 (*Jak1*, 2.6 fold, *p* < 0.01) and Janus kinase 2 (*Jak2*, 2.7 fold, *p* = 0.002) in CRS mice compared to control. A total of 49 genes were significantly induced in the *S. aureus* CRS mice compared with control (Table 1).

**Table 1:** *S. aureus* increased IFN signaling pathway in a CRS mouse model

Genbank Accession	Gene symbol	Gene name	Fold change	*p*-value
NM_009743	Bcl2l1	BCL2-like 1	2.132339	0.049560211
NM_009829	Ccnd2	Cyclin D2	2.074349	0.00042925
NM_016673	Cntfr	Ciliary neurotrophic factor receptor	0.381029	0.005855777
NM_016715	Crlf2	Cytokine receptor-like factor 2	4.247821	9.86E-05
NM_009969	Csf2	Colony stimulating factor 2 (granulocyte–macrophage)	2.424475	0.013142153
NM_009970	Csf2ra	Colony stimulating factor 2 receptor, alpha, low-affinity (granulocyte–macrophage)	5.039035	0.014062419
NM_007780	Csf2rb	Colony stimulating factor 2 receptor, beta, low-affinity (granulocyte–macrophage)	9.859299	0.003556109
NM_007781	Csf2rb2	Colony stimulating factor 2 receptor, beta 2, low-affinity (granulocyte–macrophage)	4.35353	0.000781896
NM_009971	Csf3	Colony stimulating factor 3 (granulocyte)	101.2357	0.0003478
NM_007782	Csf3r	Colony stimulating factor 3 receptor (granulocyte)	48.99682	0.000550674
NM_008337	Ifng	Interferon gamma	3.885895	0.043562027
NM_010511	Ifngr1	Interferon gamma receptor 1	2.788014	6.64E-05
NM_008338	Ifngr2	Interferon gamma receptor 2	3.017462	0.000749551
NM_010548	Il10	Interleukin 10	9.062968	0.003816983
NM_008348	Il10ra	Interleukin 10 receptor, alpha	5.012664	0.001391342
NM_008351	Il12a	Interleukin 12a	18.15763	0.000174256
NM_008353	Il12rb1	Interleukin 12 receptor, beta 1	3.749103	0.019249349
NM_133990	Il13ra1	Interleukin 13 receptor, alpha 1	2.924961	0.000435488
NM_008356	Il13ra2	Interleukin 13 receptor, alpha 2	10.69152	0.002835397
NM_172786	Il20ra	Interleukin 20 receptor, alpha	4.229001	6.36E-05
NM_021887	Il21r	Interleukin 21 receptor	3.783736	0.010314934
NM_031252	Il23a	Interleukin 23, alpha subunit p19	23.96075	0.000343771
NM_144548	Il23r	Interleukin 23 receptor	9.418073	0.001390579
AF054581	Il2ra	Interleukin 2 receptor, alpha chain	12.85514	0.002771884
NM_008368	Il2rb	Interleukin 2 receptor, beta chain	9.836039	0.002864472
NM_013563	Il2rg	Interleukin 2 receptor, gamma chain	7.242974	0.000284845
NM_008369	Il3ra	Interleukin 3 receptor, alpha chain	2.077163	0.000611051
NM_001008700	Il4ra	Interleukin 4 receptor, alpha	3.347411	0.000125086
NM_031168	Il6	Interleukin 6	26.97001	0.006988894
NM_010559	Il6ra	Interleukin 6 receptor, alpha	2.184896	0.000216122
NM_008371	Il7	Interleukin 7	2.8957	0.02218885
NM_008372	Il7r	Interleukin 7 receptor	3.433405	0.001415242
NM_008413	Jak2	Janus kinase 2	2.627008	0.009968001
NM_010589	Jak3	Janus kinase 3	2.788583	0.002084158
NM_001013365	Osm	Oncostatin M	63.33623	0.001013032
NM_011019	Osmr	Oncostatin M receptor	3.537093	0.002936044
NM_008840	Pik3cd	Phosphatidylinositol 3-kinase catalytic delta polypeptide	3.784026	0.000242313
NM_020272	Pik3cg	Phosphoinositide-3-kinase, catalytic, gamma polypeptide	4.332737	0.000944882
NM_177320	Pik3r5	Phosphoinositide-3-kinase, regulatory subunit 5, p101	4.057912	0.000583963
NM_008842	Pim1	Proviral integration site 1	10.65359	0.004996911
NM_001077705	Ptpn6	Protein tyrosine phosphatase, non-receptor type 6	2.806562	0.000924511
NM_009896	Socs1	Suppressor of cytokine signaling 1	3.82766	0.000821889
NM_007707	Socs3	Suppressor of cytokine signaling 3	8.013592	0.007643708
NM_138657	Socs7	Suppressor of cytokine signaling 7	3.028275	7.22E-05
NM_009283	Stat1	Signal transducer and activator of transcription 1	2.189129	0.041555494
AK079406	Stat3	Signal transducer and activator of transcription 3	2.498231	0.002013087
NM_011487	Stat4	Signal transducer and activator of transcription 4	3.352352	0.004112682
NM_009284	Stat6	Signal transducer and activator of transcription 6	2.093932	0.001365528
NM_009379	Thpo	Thrombopoietin	0.467665	0.00039217

Gene profiling using a gene microarray (Agilent). The significantly differentially expressed genes are listed

### SpA concentration in planktonic supernatants is increased in *S. aureus* clinical isolates from CRSwNP patients and CRSsNP patients that had multiple surgeries (> = 2 times) compared to control

**Fig. 1:**
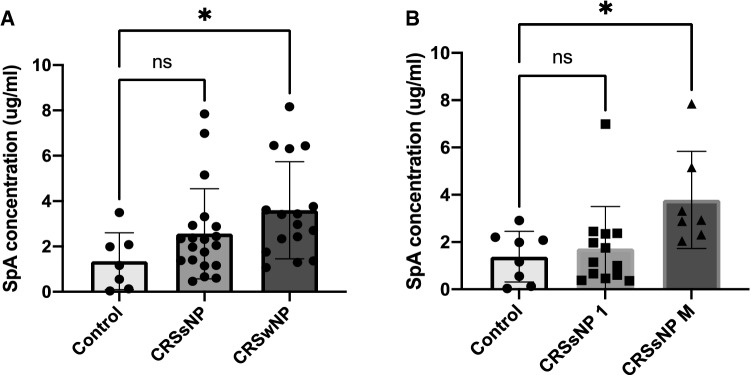
SpA content in planktonic supernatants of *S. aureus* clinical isolates. **A** represents the SpA content for all 44 *S. aureus* clinical isolates [16 CRSwNP, 20 CRSsNP, 8 controls]. **B** represents a breakdown of the CRSsNP patients into patients who has undergone 1 previous procedure CRSsNP1(*n* = 13) or multiple previous surgical procedures CRSsNPM (*n* = 7) and 8 Control patients] were grown for 24 h, conditioned media collected and SpA concentrations determined. The values are shown as means ± SEM. ns = no significant; * = *p* < 0.05, one-way ANOVA followed by Tukey’s multiple comparison post-hoc test

*S. aureus* clinical isolates from the sinonasal cavities of 16 CRSwNP, 20 CRSsNP and 8 control patients were grown for 24 h followed by SpA quantification in supernatants using ELISA based assays. Compared to control patients, SpA concentrations released by clinical isolates from CRSwNP patients were significantly increased (*p*<0.05) (Fig. 1A). The SpA concentrations released by clinical isolates from CRSsNP patients were higher in those patients that had multiple surgeries compared to controls (*p*<0.05) (Fig. 1B).

### IFNGR1 expression is increased in HNECs after SpA stimulation

**Fig. 2:**
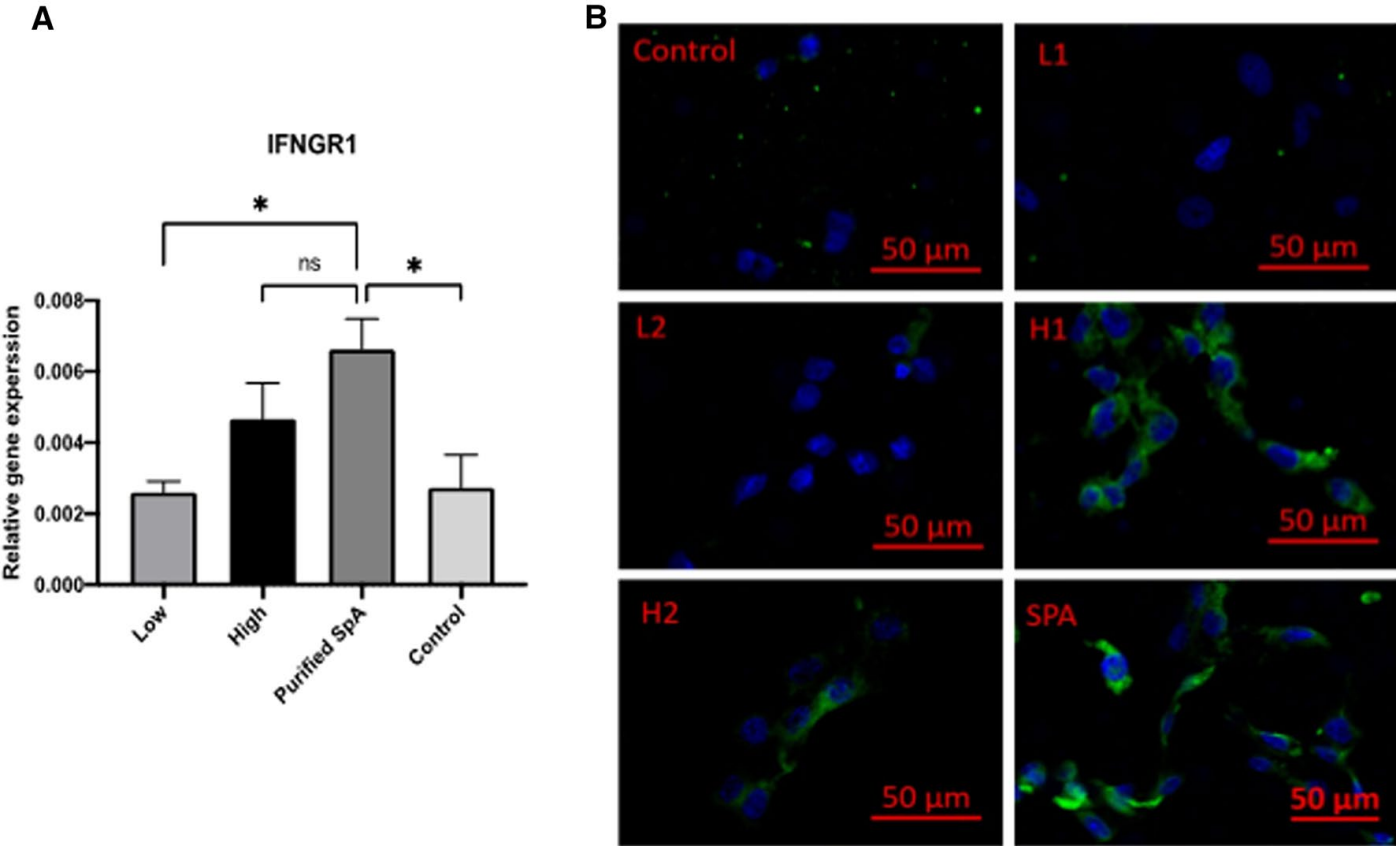
IFNGR1 expression increased in HNECs after challenge with purified SpA or *S. aureus* exoproteins containing high levels of SpA. HNECs were treated with 5% *S. aure*us planktonic supernatants (High = H1 and H2; Low = L1 and L2) and purified SpA for 4 h and 24 h, respectively followed by **A** qPCR evaluating IFNGR1 mRNA expression and **B** Immunofluorescence targeting IFNGR1 (green) and nuclei (DAPI, blue). High = *S. aureus* clinical isolates (H1, H2) having high SpA levels in planktonic supernatants; Low = *S. aureus* clinical isolates (L1, L2) having low SpA levels in planktonic supernatants; Control = 5% TSB. ns = no significant; *  *p* < 0.05

From the 44 *S. aureus* clinical isolates, we then selected those that secreted high [H1 (6.99 μg/ml) and H2 (5.16 μg/ml)] and low [L1 (0.17 μg/ml) and L2 (0.31 μg/ml)] SpA levels and harvested the planktonic supernatants after 24 h. Primary HNECs were stimulated with those supernatants or with 50 μg/ml purified SpA (positive control) or 5% TSB (negative control) for 4 h. Purified SpA significantly increased IFNGR1 mRNA expression levels compared to the low (L1 and L2) SpA supernatants and to TSB control (Fig. 2A). High (H1 and H2) SpA supernatants also increased IFNGR1 mRNA expression compared to TSB control, but this did not reach statistical significance. Immunofluorescence staining indicated the IFNGR1 expression increased in both high SpA and purified SpA treated HNECs but not low SpA treated cells or negative control (Fig. 2B).

### SpA activation of HNECs increases Jak2 phosphorylation

**Fig. 3:**
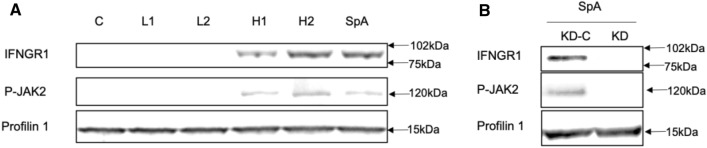
Western blot analyses of IFNGR1 and p-JAK2 protein. **A** 15 μg total protein were loaded. Anti- IFNGR1 and anti- p-JAK2 primary antibodies were applied to detect the presence of IFNGR1 and p-JAK2 protein. Profilin 1 was used as internal loading control. *C* = untreated control; H1, H2 and L1, L2 = *S. aureus* clinical isolates having high (H1, H2) and low (L1, L2) SpA concentrations in the planktonic supernatants **B** Knockdown of *Ifngr1* in HNECs followed by treatment with SpA. KD-C = lipo3000 without siRNA control, KD = *Ifgr1* gene knockdown

To investigate potential downstream SpA-dependent signaling pathways, HNECs were treated with purified SpA and supernatants isolated from planktonic *S. aureus* H1, H2 and L1, L2 and cells harvested at 6 h. Western blot showed protein bands specific for IFNGR1and p-JAK2 in cells treated with H1, H2 and purified SpA (Fig. 3A). IFNGR1 gene knockdown followed by SpA challenge did not result in an increased p-JAK2 expression (Fig. 3B). These results indicate the potential for SpA- and IFGR1-dependent JAK-2 activation in HNECs.

### SpA enhances IL-6 and IL-8 secretion by HNECs

**Fig. 4:**
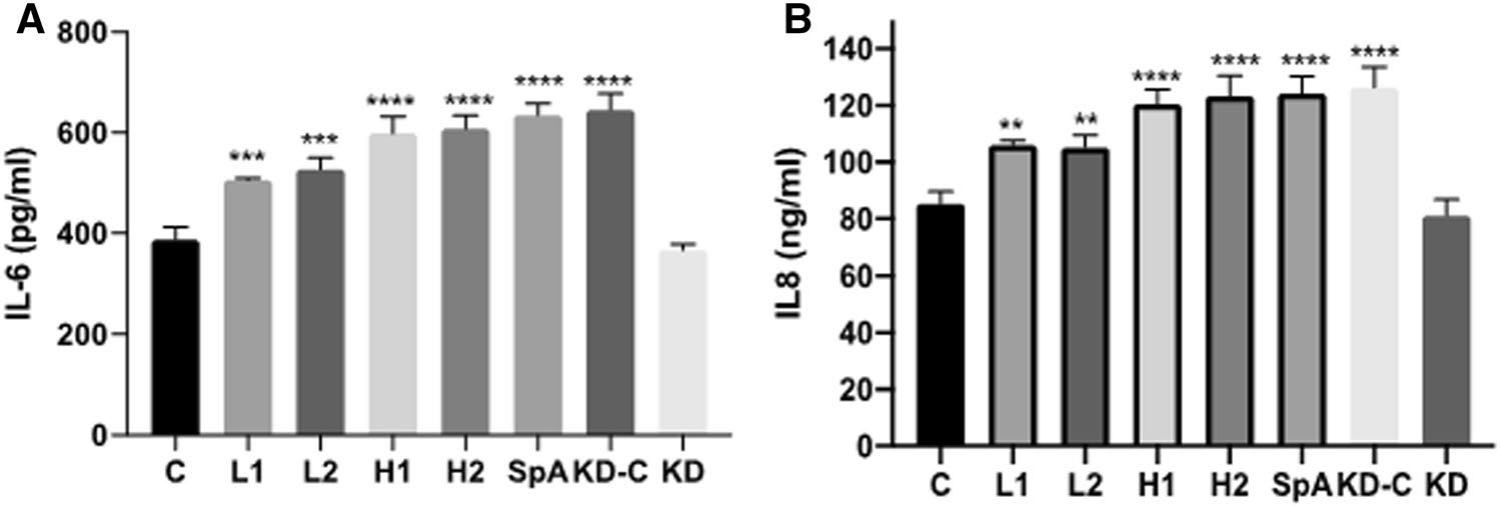
IL-6 and IL-8 secretion after challenge with SpA and bacterial supernatants. HNECs (*n* = 3 donors) were treated with SpA and *S. aureus* supernatants (H1, H2, L1, L2) for 24 h. Interleukin-6 (IL-6) **A** and Interleukin-8 (IL-8) **B** protein levels were determined with enzyme-linked immunosorbent assays (ELISA). H1, H2 and L1, L2 = *S. aureus* clinical isolates having high (H1, H2) and low (L1, L2) SpA concentrations in planktonic supernatants. *C* = untreated control, KD-C = lipo3000 without siRNA control, KD = *Ifgr*1 knockdown. The values are shown as means ± SEM. * = *P* ≤ 0.05; ** = *P* ≤ 0.01; *** = *P* ≤ 0.001; **** = *P* ≤ 0.0001, one-way ANOVA followed by Tukey’s multiple comparisons test

HNECs’ supernatants were harvested after 24 h challenge with purified SpA and the various bacterial supernatants to investigate the effect on secretion of IL-6 and IL-8. Compared with untreated Control (C) cells, both purified SpA and planktonic *S. aureus* H1 and H2 and L1 and L2 supernatants significantly increased IL-6 and IL-8 protein levels (Fig. 4), suggesting SpA and conditioned media promoted inflammatory cytokine secretion. IL-6 and IL-8 secretion was significantly higher in Purified SpA, H1 and H2 treated groups compared to control, L1 and L2 groups. However, SpA did not induce the secretion of IL-6 and IL-8 in HNECs upon *Ifgr1* knockdown.

### SpA was cytotoxic to HNECs

A significantly higher cytotoxicity was detected by LDH assays in all treatment groups compared to Control. Cytotoxicity after challenge with SpA and H2 was significantly higher than L2 and L1 challenge (SpA vs. L2: *P* = 0.0088; SpA vs. L1: *P* = 0.0096; H2 vs. L2: *P* = 0.0450; H2 vs. L1: *P* = 0.0491). H1-induced cytotoxicity was higher than L2 and L1 challenge however, no statistical significance was achieved (H1 vs. L2: *P* = 0.0621; H1 vs. L1: *P* = 0.0677). *Ifgr1* gene knockdown abrogated SpA-induced cytotoxicity (Supplementary Figure 2).

### SpA induced apoptosis of HNECs

**Fig. 5:**
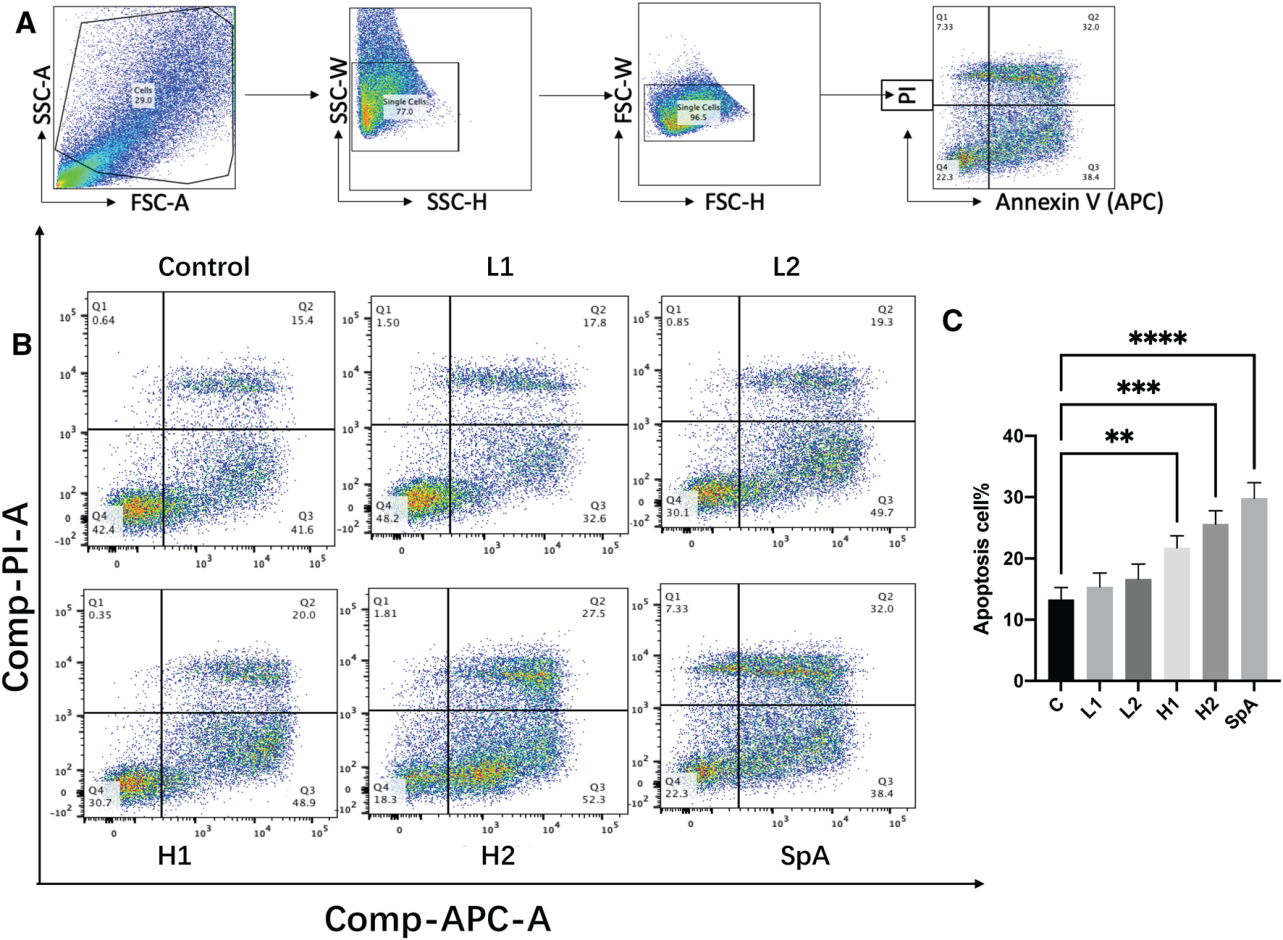
Apoptosis assay. HNECs were treated with bacterial planktonic supernatants (H1, H2, L1, L2) and purified SpA for 24 h. Cells were harvested for apoptosis assay using flow cytometry. **A** Sequential gating strategies to identify apoptosis cells; **B** Representative flow cytometry images of the percentage of apoptotic HNECs after treatment; **C** Bar graph of the percentage of apoptotic HNECs after treatment. H1, H2 and L1, L2 = S. aureus clinical isolates having high (H1, H2) and low (L1, L2) SpA concentrations in the planktonic supernatants. *C* = untreated control. The values are shown as means ± SEM. * = *P* ≤ 0.05; ** = *P* ≤ 0.01; *** = *P* ≤ 0.001; **** = *P* ≤ 0.0001, one-way ANOVA followed by Tukey’s multiple comparisons test

To determine the effect of SpA on inducing HNECs apoptosis, flow cytometry of annexin V/PI stained cells was carried out. SpA, H1 and H2 challenge for 24 h increased the frequency of apoptosis compared with Control, L2 and L1 (32%, 20% and 27.5% for SpA, H1 and H2 treated cells vs 15.4%, 17.8% and 19.3% for Control, L2 and L1 treated cells respectively) (Fig. 5A and B).

## Discussion

In this study, we observed that *S. aureus* clinical isolates from CRSsNP patients who had undergone multiple surgeries and CRSwNP patients, secreted higher amounts of SpA compared to those from non-CRS controls. Furthermore, conditioned media from isolates secreting high amounts of SpA as well as purified SpA induced the expression of IFGR1 and pJAK-2 when applied to primary human nasal epithelial cells and were more pro-inflammatory, pro-apoptotic and cytotoxic than control. A *S. aureus* CRS model showed induction of the type II interferon signaling pathway *in vivo*. Together, these findings support the notion that *S. aureus* secreted products, and in particular SpA are increased in CRSwNP patients compared to control and induce interferon signaling through activation of the IFGR1-JAK-2 pathway.

*S. aureus* is a common colonizer of the skin and nose of healthy individuals. However, *S. aureus* can also cause a range of diseases, from minor skin infections, to life-threatening pneumonia, osteomyelitis, endocarditis and sepsis. *S. aureus* is typically equipped with a plethora of virulence factors the expression of which can be modulated in response to environmental signals. This allows the bacteria to rapidly adapt to changing environments and adopt both commensal and invasive lifestyles [31]. Apart from this reversible adaptive plasticity, the results of this study indicate intrinsic genomic changes in the genetic pathways that control SpA expression and secretion in relation to CRS disease severity and phenotype [32, 33]. Indeed, increased SpA concentrations were found when *S. aureus* harvested from CRSwNP patients were grown under laboratory conditions ex vivo. Furthermore, in view of the frequent long term persistence of *S. aureus* within the sinonasal cavity of CRS patients [34] these findings indicate increased SpA secretion by *S. aureus* harvested from CRSwNP patients might reflect a pathoadaptive process that allows the bacteria to persist and dominate the sinonasal cavity concomitant with the attenuation of its virulence. Further research is required to evaluate the *in vivo* relevance of this finding and to unravel the genomic base for increased SpA expression of *S. aureus* from CRSwNP patients.

CRS can be divided into type II allergy-related CRS and non-type II CRS (EPOS2020) [35]. In both phenotypes, patients have been shown to have high levels of *S. aureus* colonization of the upper airway [36, 37]. However, *S. aureus* is also found in healthy patients and is one of the species that constitute the core microbiome of the sinonasal cavities in both CRS patients and controls [38]. Studies have found that the virulence of *S. aureus*, and in particular the presence or absence of specific toxins is associated with CRSwNP and allergic airway and skin pathologies [39, 40]. Strains of *S. aureus* that carry enterotoxins are furthermore likely to possess specific accessory gene regulator (*agr*) variants [41] and *agr* is the main regulator of staphylococcal virulence. However, this is the first study implying the potential for differences in SpA quantity that relate to CRS disease phenotype and severity. SpA is an important virulence factor of *S. aureus*, and mediates various immune evasion mechanisms via binding with host immunoglobulins thereby evading opsonophagocytic killing [24] and circumventing host immune surveillance [24]. SpA also triggers apoptotic cell death of various cell types. This includes activated B cells by linking to V_H_3 type B cell receptors [24, 42] as well as neutrophils and keratinocytes [43]. Our findings are in line with this and also found an induction of apoptosis in HNECs when challenged with purified SpA or conditioned media that contained high SpA concentrations. In contrast to necrosis, where a release of various intracellular proteins can result in a potent immune activation, cells undergoing apoptosis do not trigger inflammation and it is thought that SpA-induced apoptosis could be regarded as an immune evasion strategy [43]. At the same time, SpA also induced cytotoxic and pro-inflammatory effects stimulating the release of IL-6 and IL-8 by HNECs. Similarly, dual properties of SpA on modulating the balance of apoptotic and necrotic death pathways as well as stimulating the release of inflammatory cytokines have been reported before [43]. It can thus be postulated that the impact that *S. aureus* and SpA may have on the fate of those various cell types as well as the induction of inflammation and cytotoxicity may vary between strains and could be related to the quantity of SpA or exoproteins secreted together with SpA produced by those strains. Further research is required to elucidate the exact pathways of apoptosis or necrosis of airway epithelial cells induced by SpA and the potential dependency on expression of IFGR1.

Previous studies have shown that SpA can signal through the TNF-α receptor 1 (TNFR1) in various cell types including airway epithelial cells [44], monocytes/macrophages and neutrophils [45]. TNFR1 activation has a central role in initiating both pro-inflammatory and pro-apoptotic signaling cascades in those cells [43]. SpA has also been shown to activate type I IFN signaling [26] and such responses have recently been shown to contribute to the pathophysiology of eosinophilic CRS [27]. Type I interferons along with various cytokines including IL-12, IL-15 and IL-18 can induce IFN-γ production from natural killer (NK) cells and once released, IFN-γ signals through the IFNGR [46]. Our findings add to this and indicate that SpA can induce IFGR1 expression and activates IFGR1 dependent JAK-2 signaling in HNECs. Given that IFGR1 knockdown abrogated SpA-dependent JAK2 phosphorylation, cytotoxicity and pro-inflammatory effects, it is postulated that those effects are at least in part dependent on activation of the IFGR in HNECs. Further research is needed to evaluate whether SpA can activate the IFGR directly or whether the induction of its expression and subsequent activation is secondary and dependent on specific cytokines such as IFN-γ.

## Supplementary Information

Below is the link to the electronic supplementary material.

Supplementary file1 (DOCX 5512 KB)

Supplementary file2 (DOCX 62 KB)

## References

[1] Simon A, Dresbach T, Muller A (2018). Methicillin-resistant *Staphylococcus* aureus decolonization in neonates and children. Pediatr Infect Dis J.

[2] van Hal SJ, Jensen SO, Vaska VL, Espedido BA, Paterson DL, Gosbell IB (2012). Predictors of mortality in *Staphylococcus* aureus bacteremia. Clin Microbiol Rev.

[3] Bassetti M, Righi E, Del Giacomo P, Sartor A, Ansaldi F, Trucchi C (2018). Predictors of mortality with *Staphylococcus* aureus bacteremia in elderly adults. J Am Geriatr Soc.

[4] Fischbach MA, Walsh CT (2009). Antibiotics for emerging pathogens. Science.

[5] Klevens RM, Edwards JR, Tenover FC, McDonald LC, Horan T, Gaynes R (2006). Changes in the epidemiology of methicillin-resistant Staphylococcus aureus in intensive care units in US hospitals, 1992–2003. Clin Infect Dis.

[6] Delaney JAC, S-L V, Brassard P, Suissa S (2008). Mortality after infection with methicillin-resistant *Staphylococcu*s aureus (MRSA) diagnosed in the community. BMC Med.

[7] Cegelski L, Marshall GR, Eldridge GR, Hultgren SJ (2008). The biology and future prospects of antivirulence therapies. Nat Rev Microbiol.

[8] Rasko DA, Sperandio V (2010). Anti-virulence strategies to combat bacteria-mediated disease. Nat Rev Drug Discov.

[9] Hanke ML, Heim CE, Angle A, Sanderson SD, Kielian T (2013). Targeting macrophage activation for the prevention and treatment of *Staphylococcus* aureus biofilm infections. J Immunol.

[10] Melehani JH, James DB, DuMont AL, Torres VJ, Duncan JA (2015). Staphylococcus aureus leukocidin A/B (LukAB) kills human monocytes via host NLRP3 and ASC when extracellular, but not intracellular. PLoS Pathog.

[11] Jacobs SA, Diem MD, Luo J, Teplyakov A, Obmolova G, Malia T (2012). Design of novel FN3 domains with high stability by a consensus sequence approach. Protein Eng Des Sel.

[12] Odegrip R, Coomber D, Eldridge B, Hederer R, Kuhlman PA, Ullman C (2004). CIS display: in vitro selection of peptides from libraries of protein-DNA complexes. Proc Natl Acad Sci U S A.

[13] Barczak AK, Hung DT (2009). Productive steps toward an antimicrobial targeting virulence. Curr Opin Microbiol.

[14] Muhlen S, Dersch P (2016). Anti-virulence strategies to target bacterial infections. Curr Top Microbiol Immunol.

[15] Kobayashi N, Urasawa S, Uehara N, Watanabe N (1999). Analysis of genomic diversity within the Xr-region of the protein a gene in clinical isolates of *Staphylococcus* aureus. Epidemiol Infect.

[16] Seldon TA, Hughes KE, Munster DJ, Chin DY, Jones ML (2011). Improved protein-A separation of VH3 Fab from Fc after papain digestion of antibodies. J Biomol Techn JBT.

[17] Foster TJ (2005). Immune evasion by staphylococci. Nat Rev Microbiol.

[18] Foster TJ (2009). Colonization and infection of the human host by staphylococci: adhesion, survival and immune evasion. Vet Dermatol.

[19] Veldkamp KE, van Strijp JA (2009). Innate immune evasion by staphylococci. Adv Exp Med Biol.

[20] Viau M, Longo NS, Lipsky PE, Zouali M (2005). Staphylococcal protein a deletes B-1a and marginal zone B lymphocytes expressing human immunoglobulins: an immune evasion mechanism. J Immunol.

[21] Martin F, Kearney JF (2002). Marginal-zone B cells. Nat Rev Immunol.

[22] Paciorkowski N, Porte P, Shultz LD, Rajan T (2000). B1 B lymphocytes play a critical role in host protection against lymphatic filarial parasites. J Exp Med.

[23] Kreuk LS, Koch MA, Slayden LC, Lind NA, Chu S, Savage HP (2019). B cell receptor and toll-like receptor signaling coordinate to control distinct B-1 responses to both self and the microbiota. Life.

[24] Falugi F, Kim HK, Missiakas DM, Schneewind O (2013). Role of protein A in the evasion of host adaptive immune responses by *Staphylococcus* aureus. MBio.

[25] Lee AJ, Ashkar AA (2018). The dual nature of type I and type II interferons. Front Immunol.

[26] Martin FJ, Gomez MI, Wetzel DM, Memmi G, O'Seaghdha M, Soong G (2009). *Staphylococcus* aureus activates type I IFN signaling in mice and humans through the Xr repeated sequences of protein A. J Clin Invest.

[27] Jang YJ, Lim JY, Kim S, Lee Y, Kweon MN, Kim JH (2018). Enhanced interferon-beta response contributes to eosinophilic chronic rhinosinusitis. Front Immunol.

[28] Parker D, Planet PJ, Soong G, Narechania A, Prince A (2014). Induction of type I interferon signaling determines the relative pathogenicity of *Staphylococcus* aureus strains. PLoS Pathog.

[29] Mullol B, Douglas G, Hopkins K (2012). EPOS 2012: European position paper on rhinosinusitis and nasal polyps 2012. Rhinology.

[30] Jacob A, Faddis BT, Chole RA (2001). Chronic bacterial rhinosinusitis: description of a mouse model. Arch Otolaryngol Head Neck Surg.

[31] Balasubramanian D, Harper L, Shopsin B, Torres VJ (2017). *Staphylococcus* aureus pathogenesis in diverse host environments. Pathog Dis.

[32] Schneewind O, Missiakas D (2014). Sec-secretion and sortase-mediated anchoring of proteins in gram-positive bacteria. Biochim Biophys Acta.

[33] Cheung AL, Eberhardt K, Heinrichs JH (1997). Regulation of protein a synthesis by the sar and agr loci of *Staphylococcus* aureus. Infect Immun.

[34] Drilling A, Coombs GW, Tan HL, Pearson JC, Boase S, Psaltis A (2014). Cousins, siblings, or copies: the genomics of recurrent *Staphylococcus* aureus infections in chronic rhinosinusitis. Int Forum Allergy Rhinol.

[35] Fokkens WJ, Lund VJ, Hopkins C, Hellings PW, Kern R, Reitsma S (2020). European position paper on rhinosinusitis and nasal polyps 2020. Rhinology.

[36] Suh JD, Ramakrishnan V, Palmer JN (2010). Biofilms. Otolaryngol Clin North Am.

[37] Vickery TW, Ramakrishnan VR, Suh JD (2019). The role of *Staphylococcus* aureus in patients with chronic sinusitis and nasal polyposis. Curr Allergy Asthma Rep.

[38] Paramasivan S, Bassiouni A, Shiffer A, Dillon MR, Cope EK, Cooksley C (2020). The international sinonasal microbiome study: a multicentre, multinational characterization of sinonasal bacterial ecology. Allergy.

[39] Nakamura Y, Oscherwitz J, Cease KB, Chan SM, Munoz-Planillo R, Hasegawa M (2013). *Staphylococcus* delta-toxin induces allergic skin disease by activating mast cells. Nature.

[40] Teufelberger AR, Broker BM, Krysko DV, Bachert C, Krysko O (2019). *Staphylococcus* aureus orchestrates type 2 airway diseases. Trends Mol Med.

[41] Van Zele T, Vaneechoutte M, Holtappels G, Gevaert P, van Cauwenberge P, Bachert C (2008). Detection of enterotoxin DNA in staphylococcus aureus strains obtained from the middle meatus in controls and nasal polyp patients. Am J Rhinol.

[42] Goodyear CS, Silverman GJ (2003). Death by a B cell superantigen: in vivo VH-targeted apoptotic supraclonal B cell deletion by a staphylococcal toxin. J Exp Med.

[43] Ledo C, Gonzalez CD, Garofalo A, Sabbione F, Keitelman IA, Giai C (2020). Protein a modulates neutrophil and keratinocyte signaling and survival in response to *Staphylococcus* aureus. Front Immunol.

[44] Gomez MI, Lee A, Reddy B, Muir A, Soong G, Pitt A (2004). Staphylococcus aureus protein a induces airway epithelial inflammatory responses by activating TNFR1. Nat Med.

[45] Giai C, Gonzalez CD, Sabbione F, Garofalo A, Ojeda D, Sordelli DO (2016). *Staphylococcus* aureus induces shedding of IL-1RII in monocytes and neutrophils. J Innate Immun.

[46] Pegram HJ, Andrews DM, Smyth MJ, Darcy PK, Kershaw MH (2011). Activating and inhibitory receptors of natural killer cells. Immunol Cell Biol.

