# Supplementary material for: Staphylococcal protein A modulates inflammation by inducing interferon signaling in human nasal epithelial cells
Source: Inflamm Res. 2022 Dec 17;72(2):251–62. doi: 10.1007/s00011-022-01656-1 (PMC9925485; doi:10.1007/s00011-022-01656-1)
Supplement: Supplementary file 1 — Supplementary file1 (DOCX 5512 KB) [file 11_2022_1656_MOESM1_ESM.docx]

**Supplementary Figure 1: H&E staining of the middle turbinate nasal mucosa in control and *S. aureus* CRS mouse model.**

Hematoxylin and eosin staining of control sinonasal tissues (A, B) and *S. aureus* CRS (C, D). Imaging using a Leica light microscope system. A and C: Control and CRS mice sections imaged with Low magnification. B and D: Control and CRS mice sections imaged with high magnification. *showed ciliary denudation, ↗showed lymphocyte.
