# Supplementary material for: Staphylococcal protein A modulates inflammation by inducing interferon signaling in human nasal epithelial cells
Source: Inflamm Res. 2022 Dec 17;72(2):251–62. doi: 10.1007/s00011-022-01656-1 (PMC9925485; doi:10.1007/s00011-022-01656-1)
Supplement: Supplementary file 2 — Supplementary file2 (DOCX 62 KB) [file 11_2022_1656_MOESM2_ESM.docx]

**Supplementary Figure 2: Cytotoxicity of HNECs challenged with SpA and *S. aureus* supernatants.**

HNECs were treated with 5% bacterial planktonic supernatants (H1, H2, L1 and L2) and purified SpA for 24 hours followed by lactate dehydrogenase assays to determine the cytotoxicity. H1, H2 and L1, L2= *S. aureus* clinical isolates having high (H1, H2) and low (L1, L2) SpA concentrations in the planktonic supernatants. C=5% tryptic soy broth (TSB) in medium, positive control = 10% Triton X-100 in medium, KD-C= lipo3000 without siRNA control, KD= *Ifgr1* knockdown. The values are shown as means ± SEM. *=P ≤ 0.05; ***=P ≤ 0.001; ****= P ≤ 0.0001 using one-way ANOVA followed by Tukey’s multiple comparisons test.
